# Experimental and theoretical study of donor-π-acceptor compounds based on malononitrile

**DOI:** 10.1186/s13065-018-0394-5

**Published:** 2018-03-09

**Authors:** Mohie E. M. Zayed, Reda M. El-Shishtawy, Shaaban A. Elroby, Khalid O. Al-Footy, Zahra M. Al-amshany

**Affiliations:** 1 Chemistry Department, Faculty of Science, King Abdulaziz University, P. O. Box 80203, Jeddah, Saudi Arabia; 2 Dyeing, Printing and Textile Auxiliaries Department, Textile Research Division, National Research Center, Dokki, Cairo, 12622 Egypt; 3 Chemistry Department, Faculty of Science, Beni-Suef University, Beni-Suef, 6251 Egypt

**Keywords:** Donor-π-acceptor, Dicyanovinyl, UV–visible and fluorescence spectra, Molecular rotor, DFT, TD-DFT

## Abstract

A set of different donor-π-acceptor compounds having dicyanovinyl as the acceptor and aryl moieties as donors were synthesized by Knoevenagel condensation. The UV–visible absorption and fluorescence spectra were investigated in different solvents. The optical band gab energy (Eg) was linearly correlated with the Hammett resonance effect of the donor to reveal that the higher the value of Hammett resonance effect of a donor, the lower the Eg of the molecule. The photophysical data revealed that compounds M4–M6 are typical molecular rotors with fluorescence due to twisted intramolecular charge transfer. Compound M5 revealed the largest Stokes shift (11,089 cm^−1^) making it a useful fluorescent sensor for the changes of the microenvironment. The effect of substituents on the optical properties of donor-π-acceptor compounds having dicyanovinyl as the acceptor are studied using density functional theory and time-dependent density functional theory (DFT/TD-DFT). The optical transitions are thoroughly examined to reveal the impact of subtituents on both absorption and fluorescence, mainly through the modification of the structure in the excited state. The theoretical results have shown that TD-DFT calculations, with a hybrid exchange–correlation and the long-range corrected density functional PBEPBE with a 6–311++G** basis set, was reasonably capable of predicting the excitation energies, the absorption and the emission spectra of these molecules.

## Introduction

Donor-π-conjugate-electron acceptor (D-π-A) compounds are characterized by having intramolecular charge transfer (ICT) character. These compounds are of great interest owing to their high molar absorptivity [1], amenability of tuning their color by changing the donor, acceptor, and/or π linker [2, 3] and potential applications in optoelectronics [4–6], sensors [7, 8], solvent polarity and others [9]. It is known that cyano group is one of the strongest attracting groups and has been used for the construction of D-π-A dyes [10–20]. On the other hand, dimethylamino group is a strong electron donating group compared with methoxy and/or methyl group.

In this context, we have designed and prepared as series of different benzenoid compounds containing different numbers of methoxy groups, methyl group and dimethylamino group as electron donors compared with the unsubstantiated benzene ring and using dicyanovinyl as the electron acceptor group. It was hypothesized that having acceptor in one side of a conjugated system and connected with different donors on the other side would help understanding the ICT character of such compounds and its impact in their photophysical properties.

In recent years, calculations of electronic structures in the excited states have been a focus of interest because of the development of computations based on Gaussian and the time dependent density functional theory (TDDFT) [21–23]. Also, the solvent effect on the electronic absorption spectra is a useful tool to identify the electronic transitions of the molecules. This would help in studying the chemical properties of the excited states and to distinguish between the different electronic transitions. We will use the Continuum Polarizable model (PCM) [24, 25].

Therefore, computational chemistry is thus necessary to get insight into the molecular structure, although according to our best knowledge no evidence of similar study for the dicyanovinyl effect on the ICT character of the model compounds selected in this study. In this work, interest resides in correlating the theoretically predicted electronic parameters with the accurate experimental results so as to provide possible explanations for the experimentally observed data.

## Results and discussion

### Synthesis

**Scheme 1:**
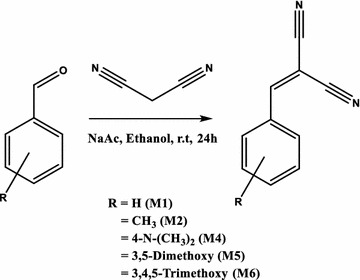
Synthesis of molecular rotors

The compounds (M1, M2, M4–6) were obtained by Knoevenagel condensation in a basic medium as shown in Scheme 1. The structure of these compounds was confirmed by 1H and 13C NMR, mass spectrometry and FTIR.

### UV–Visible and fluorescence spectra

**Fig. 1:**
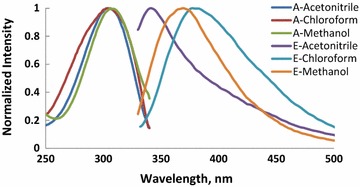
Normalized absorption (A) and emission (E) spectra of compound M1 (1 × 10^−5 ^M) in different solvents

**Fig. 2:**
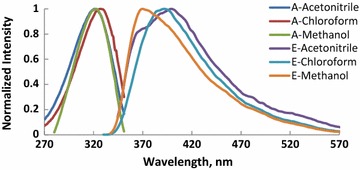
Normalized absorption (A) and emission (E) spectra of compound M2 (1 × 10^−5 ^M) in different solvents

**Fig. 3:**
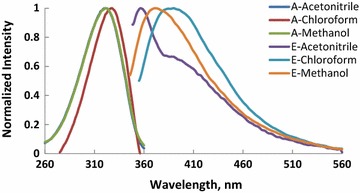
Normalized absorption (A) and emission (E) spectra of compound M3 (1 × 10^−5 ^M) in different solvents

**Fig. 4:**
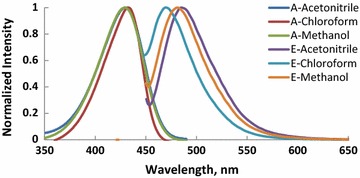
Normalized absorption (A) and emission (E) spectra of compound M4 (1 × 10^−5 ^M) in different solvents

**Fig. 5:**
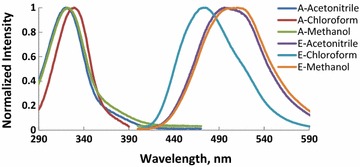
Normalized absorption (A) and emission (E) spectra of compound M5 (1 × 10^−5 ^M) in different solvents

**Fig. 6:**
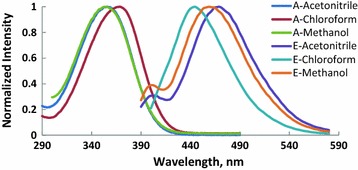
Normalized absorption (A) and emission (E) spectra of compound M6 (1 × 10^−5 ^M) in different solvents

Absorption and fluorescence spectra of molecules (M1–6) recorded in CHCl_3_, CH_3_OH and CH_3_CN and the photophysical properties of these compounds are shown in Figs. 1, 2, 3, 4, 5, 6 and summarized Table 1, respectively. The molar absorptivity of these compounds indicates that their electronic transition is due to π–π^*^. The effect of the donor ability of the substituent groups is nicely correlated with the optical data. Substituting hydrogen atom in compound 1 with different donors shown in Scheme 1 results in a bathochromic shifts in the absorption and in accordance with the donor ability of the substituents.

**Table 1:** Photophysical data of compounds M1–6 in different solvents

M	Chloroform				Methanol				Acetonitrile			
	ε, M^−1^ cm^−1^ × 104	λ_abs_	λ_em_	Stokes shift, cm^−1^	ε, M^−1^ cm^−1^ × 104	λ_abs_	λ_em_	Stokes shift, cm^−1^	ε, M^−1^ cm^−1^ × 104	λ_abs_	λ_em_	Stokes shift, cm^−1^
M1	2.35	305	377	6262	3.16	306	370	5653	2.35	304 (324)^a^	341	3569
M2	2.92	327	392	5071	2.20	323	370	3933	2.76	321 (348)	399	6090
M3	3.05	327	390	4940	3.23	321	372	4271	3.06	321 (434)	357	3141
M4	6.02	432	470	1872	5.77	429	481	2520	5.53	430 (403)	485	2637
M5	1.86	329	475	9343	1.86	322	511	11,486	1.92	320 (319)	496	11,089
M6	2.24	369	445	4628	2.16	353	459	6542	2.03	356 (437)	469	6768

^a^Data in brackets are theoretical values using PBEPBE/6–311++G** level of theory in acetonitrile solvent

**Fig. 7:**
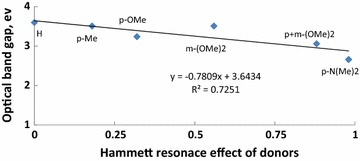
Hammett resonance effect of donors versus optical band gap of compounds M1–6

As the donor groups are in conjunction with acceptor via π-system, thus it was reasonable to correlate the calculated band gap energy of all compounds with Hammett resonance effect [26]. The optical band gap (E_g_) was estimated from the onset wavelength of absorption using the equation of E_g_ = 1240/λ_ab_, onset. Figure 7 shows a linear relation between E_g_ and Hammett resonance effect of donors. As shown in this figure, the higher the value of Hammett resonance effect of a donor, the lower the E_g_ of the molecule indicating the involvement of an intramolecular charge transfer (ICT) between donor and acceptor.

**Fig. 8:**
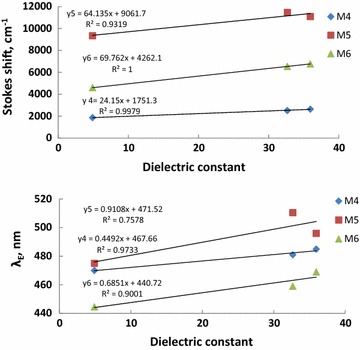
Solvent polarity versus Stokes shift and emission wavelengths of compounds M4–6

**Fig. 9:**
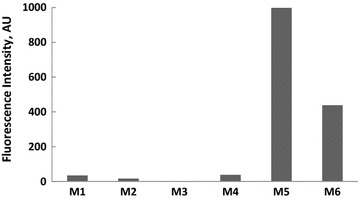
Relative fluorescence intensity of compounds M1-6 in acetonitrile (1 × 10^−5 ^M)

Another interesting feature observed in Table 1 and Figs. 1, 2, 3, 4, 5, 6 is the enhanced Stokes shift and bathochromic shift of emission for in different solvents. Correlating the solvents polarity in terms of their dielectric constants with Stokes shifts and emission wavelengths of M4–6 (Fig. 8) gives a direct linear proportion indicating that compounds M4–6 are typical molecular rotors. Molecular rotors are donor-π-acceptor compounds that emit as a result of twisted intramolecular charge transfer (TICT) due to the rotation of donor and/or acceptor in the ground and excited states around sigma bond [27]. This TICT is greatly manifested in compound M5 as evidenced by its relatively higher fluorescence intensity (Fig. 9) as well as its largest Stokes shift (Table 1).

The fluorescent intensity is a function of the free rotation of the molecular rotor and thus a higher fluorescence would be observed dependent on the nature of TICT and/or the fluorophore microenvironment. Since the solvents used are non-viscous solvents thus the huge fluorescence observed in compound M5 compared with other compounds is reflecting its twisted geometry that hampers the free rotation. It is worth noting (Table 1) that compound M5 has the lowest molar absorptivity among all compounds studied indicating a relatively twisted ground state. The very large Stokes shift observed in compound M5 is of practical usefulness as such property would reduce the overlap between the UV–vis absorption and emission spectra of the compound and consequently minimizing the so-called inner filter effect and thus rendering compound M5 as an environment-sensitive fluorescent probe [9, 28–30].

### Molecular orbital calculations

**Fig. 10:**
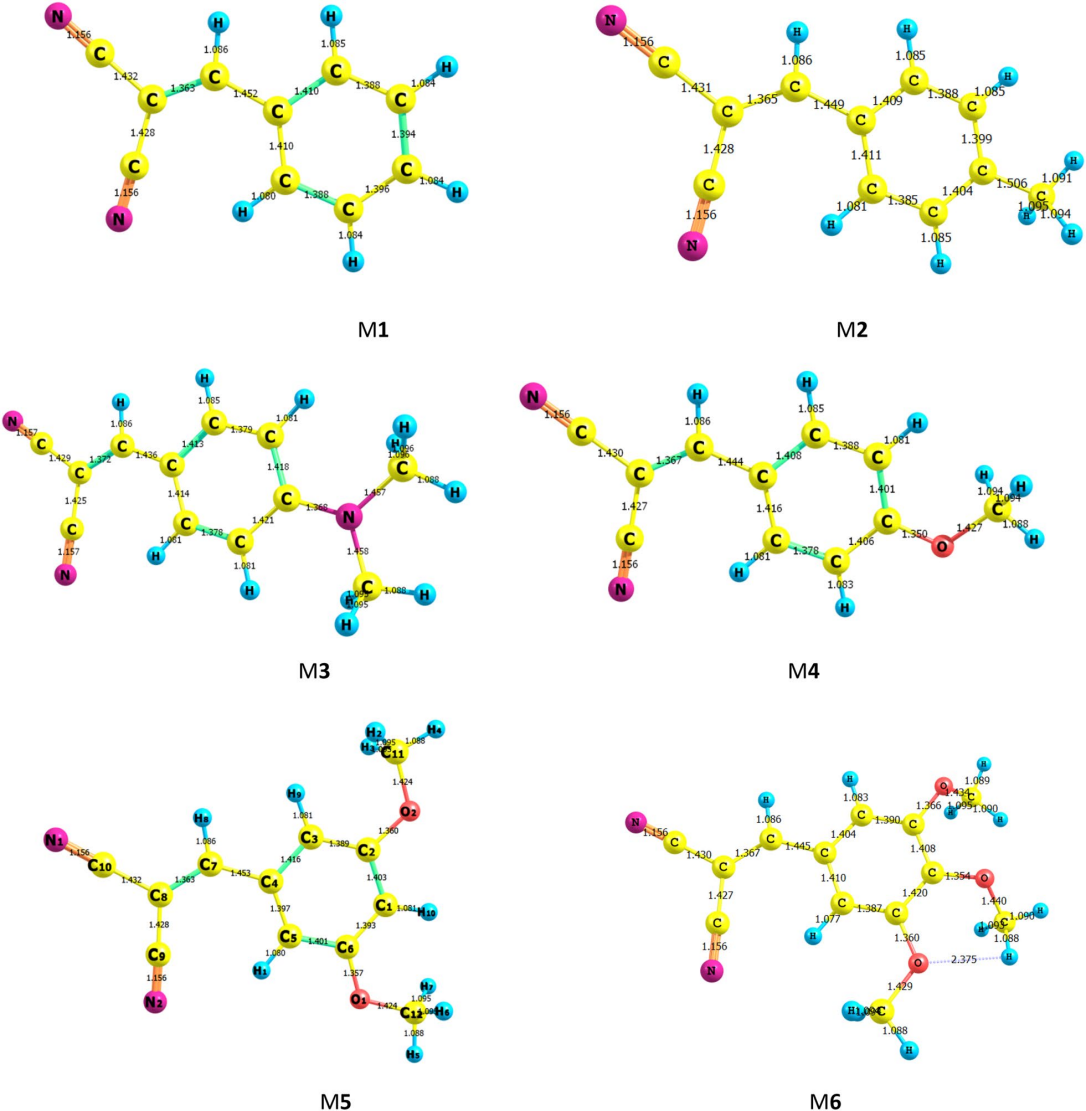
Optimized geometries (bond lengths/Ǻ) of the ground state for the studied compounds using B3LYP/6–311++G** level of theory

**Fig. 11:**
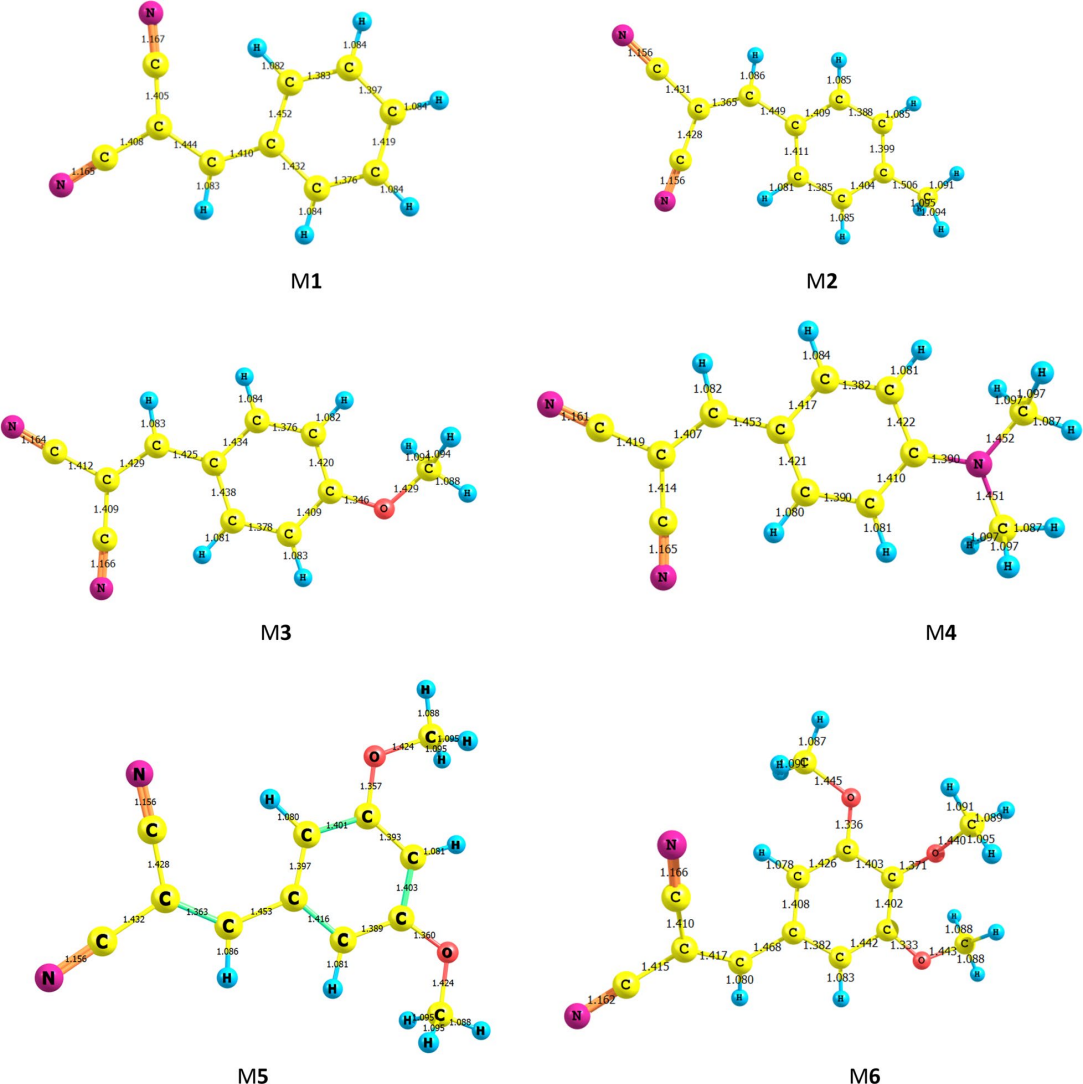
Optimized geometries (bond lengths/Ǻ) of the excited state for studied compounds using B3LYP/6–311++G** level of theory

The optimized geometries obtained by B3LYP/6-311++G** level of theory for the ground and excited states studied molecules are displayed in Figs. 10 and 11, respectively. DFT calculations give planar optimal geometries for ground and excited states. The characterization of the delocalization of π-electrons along the molecule can be estimated by the difference between single and double bond lengths. The small difference between single and double bond lengths corresponds to delocalized charge density on all over the molecules. Table 2 shows the bond lengths and differences between single and double bonds for ground and excited states of the optimal geometries obtained using B3LYP/6-311++G** level of theory. The difference between C–C and C=C in M3 and M5 decrease compared to the other compounds in both ground and excited states. This result indicated that π electron density becomes stronger upon photoexcitation. The bonds between donor and acceptor groups are C8-C1 and C8=C9. The shorter length of these bonds favored the charge transfer (CT) within the studied molecules. Table 2 shows that C8=C9 of M1, M2, M3, M4, M5 and M6 are 1.363, 1.365, 1.372, 1.367, 1.369 and 1.367 Å respectively, while C8-C1 shows more single C–C features. The difference between double and single bond lengths are sorted in the order of M5 > M4 > M6 > M2 > M1, which presents the intensity of interaction between donor and acceptor groups. For all the studied molecules, C8-C1 does not change significantly. The difference between double and single bond lengths are significantly decreased for the excited state (S1) compared to those in the ground state (S_0_), especially in M3 and M5 molecules. These results indicate that the connection between acceptor group and donor group for highly enhanced ICT character, which is important for the absorption spectra red-shift.

**Table 2:** Optimized Selected Bond lengths of the studied molecules obtained by B3LYP/6–311++G** level

M	Ground state			Excited state	
	C4–C8	C8–C9	(4-C9)-(8-C9)	C4-C8	C8-C9
M1	1.452	1.363	0.089	1.410	1.444
M2	1.449	1.365	0.084	1.365	1.449
M3	1.436	1.372	0.064	1.458	1.407
M4	1.444	1.367	0.077	1.425	1.429
M5	1.453	1.363	0.090	1.4507	1.417
M6	1.445	1.367	0.078	1.468	1.417

#### Absorption spectra

**Fig. 12:**
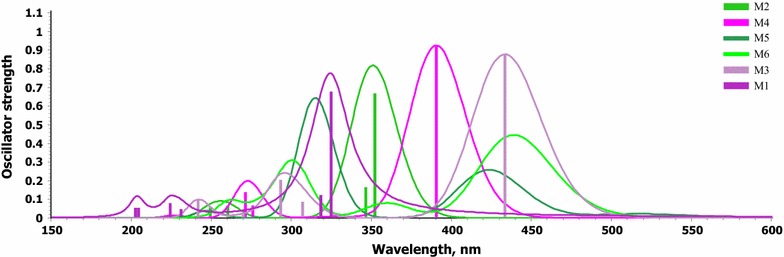
The UV–visible absorption spectra of the studied compounds calculated using PBEPBE/6–311++G** level of theory in chloroform

**Fig. 13:**
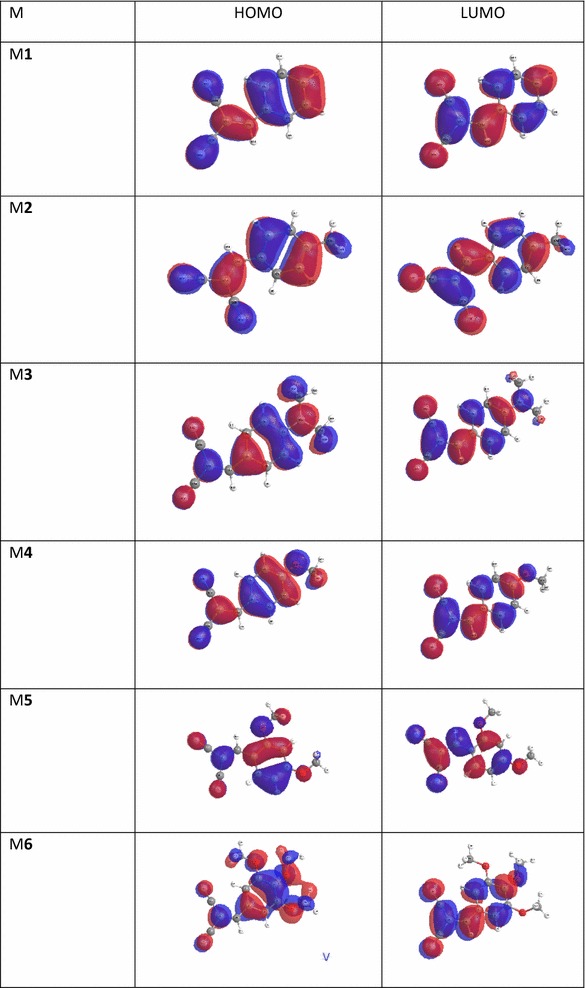
Schematic diagram of NTO’s of four studied dyes calculated at the PBEPBE/6–311++G∗∗ level of theory. The surfaces are generated with an isovalue at 0.02

The vertical excited first three singlet states, transitions energies, and oscillator strength using TD-DFT (PBEPBE) method started from the optimized structures have been calculated. The corresponding simulated UV–visible absorption spectra of all molecules in the gas phase using PBEPBE/6-311++G** level of theory displays in Fig. 12. Table 3 reveals the calculated absorption λ_max_ (nm), frontier molecular orbitals contributions and oscillator strength (f) of the studied compounds (M) collected in Table 3. As shown in Fig. 13 and Table 3, all compounds exhibit a strong absorption band in the region around 450 − 200 nm, which can be assigned to an intramolecular charge transfer (ICT) between the various donating unit and the electron acceptor groups. The λ_abs_ of the studied molecules decreases in the following order M6 > M3 > M5 > M4 > M2 > M1 which is the same order of the band gap except with M3. This bathochromic effect from M1 (304.27 nm) to M3 (397.62) is obviously due to increased π delocalization. With the increasing of conjugation, the λ_abs_ arising from S_0_ → S_1_ electronic transition increase. The first excited states for all studied molecules are π → π∗ transitions which differ in the dominant configuration. The natural transition orbitals (NTO) displayed in Fig. 13, which indicate that all transitions are of π → π∗ and have a pronounced charge-transfer character. HOMO and LUMO show a pronounced electronic density shift from the donor to the acceptor groups.

**Table 3:** Absorption wavelength (nm), molecular orbital contribution, energy level of HOMO, LUMO and oscillator strength calculated by using PBEPBE/6–311 ++G** level of theory in gas phase

M	Wave length (nm)	f	MO contribution	MO coeff. (%)
M1	304	0.542	HOMO–LUMO	94
	297	0.119	HOMO-1-LUMO	91
	179	0.414	HOMO-1-LUMO+1	57
M2	331	0.536	HOMO–LUMO	85
	335	0.15	HOMO-1-LUMO	84
M3	397	0.705	HOMO–LUMO	98
	285	0.216	HOMO-1-LUMO	89
M4	354	0.69	HOMO–LUMO	98
	269	0.147	HOMO-2-LUMO	71
M5	393	0.154	HOMO-1-LUMO	63
	300	0.519	HOMO-2-LUMO	62
M6	434	0.17	HOMO-1-LUMO	96
	413	0.339	HOMO–LUMO	92
	294	0.289	HOMO-3-LUMO	79

## Experimental section

### General

All solvents and reagents were purchased from Sigma-Aldrich Company and used as received. 2-(4-Methoxybenzylidene)malononitrile (M3) is commercially available at Life Chemicals, Canada and was used as received. 1H and 13C NMR spectra were recorded in CDCl_3_ solutions on a Bruker Avance 600 MHz spectrometer. Infrared spectra were performed on a PerkinElmer spectrum 100 FTIR spectrometer. Mass spectra were measured on a GCMS-QP1000 EX spectrometer at 70 eV. UV–visible absorption spectra were recorded with a Jasco V560 spectrophotometer (Jasco international Co., Ltd., Tokyo, Japan). Fluorescence spectra were conducted on a Perkin-Elmer LS-55 Luminescence Spectrometer and uncorrected. Melting points were determined in open capillary tubes in a Stuart Scientific melting point apparatus SMP3 and are uncorrected.

### Synthesis

#### General procedure

A mixture of aldehyde derivative (10 mmol), malononitrile (10 mmol), sodium acetate anhydrous (12 mmol) and ethanol absolute (30 ml) were stirred at room temperature for 24 h. Then, water was added to the reaction mixture to precipitate the product. The precipitate was filtered, washed water and then dried. Further purification by silica gel column chromatography afforded the corresponding product in good yield.

##### 2-Benzylidenemalononitrile (M1)

Solid, m.p:84 °C ^1^H NMR (600 MHz, CDCl_3_): ∂ 7.54 (t, 2H, J = 7.2 Hz, Ar–CH), 7.63 (t, 2H, J = 7.2 Hz, Ar–CH), 7.78 (s, 1H, CH=(CN)_2_), 7.90 (d, 2H, J = 7.2 Hz, Ar–CH). ^13^C NMR (150 MHz, CDCl_3_): ∂ 82.88, 112.57, 113.73, 129.67, 130.77, 130.93, 134.69, 159.9; ATR-IR: 3043, 2222, 1589, 1567, 1449; MS (*m/z*) for C_10_H_6_N_2_ (M−H)^+^: Calcd: 153.05, Found: 153.

##### 2-(4-Methylbenzylidene)malononitrile (M2)

Solid, m.p:135 °C ^1^H NMR (600 MHz, CDCl_3_): ∂ 2.45 (s, 3H, CH_3_), 7.32 (d, 2H, J = 7.8 Hz, Ar–CH), 7.71 (s, 1H, CH=(CN)_2_), 7.8 (d, 2H, J = 7.8 Hz, Ar–CH). ^13^C NMR (150 MHz, CDCl_3_): ∂ 22.05, 81.27, 112.88, 114.04, 128.50, 130.41, 130.95, 146.41, 159.79; ATR-IR: 3035, 2221, 1605, 1584, 1553, 1509; MS (*m/z*) for C_11_H_8_N_2_ (M−H)^+^: Calcd: 167.07, Found: 167.

##### 2-(4-(Dimethylamino)benzylidene)malononitrile (M4)

Solid, m.p:180 °C ^1^H NMR (600 MHz, CDCl_3_): ∂ 3.14 (s, 6H, N(CH_3_)_2_), 6.68 (d, 2H, J = 9 Hz, Ar–CH), 7.46 (s, 1H, CH=(CN)_2_), 7.81 (d, 2H, J = 9 Hz, Ar–CH). ^13^C NMR (150 MHz, CDCl_3_): ∂ 40.15, 71.95, 111.60, 114.95, 116.03, 119.31, 133.83, 154.22, 158.16; ATR-IR: 2920, 2207, 1607, 1560, 1515, 1385, 1357; MS (*m/z*) for C_12_H_11_N_3_ (M−H)^+^: Calcd: 196.1, Found: 196.

##### 2-(3,5-Dimethoxybenzylidene)malononitrile (M5)

Solid, m.p:89 °C ^1^HNMR (600 MHz, CDCl_3_): ∂ 3.83 (s, 6H, OCH_3_), 6.69 (s, 1H, Ar–CH), 7.03 (s, 2H, Ar–CH), 7.68 (s, 1H, CH=(CN)_2_).^13^C NMR (150 MHz, CDCl_3_): ∂ 55.71, 83.12, 107.22, 108.24, 112.66, 113.68, 132.35, 160.14, 161.25; ATR-IR: 2966, 2229, 1603, 1577, 1458, 1426, 1310; MS (*m/z*) for C_12_H_10_N_2_O_2_ (M−H)^+^: Calcd: 213.07, Found: 213.

##### 2-(3,4,5-Trimethoxybenzylidene)malononitrile (M6)

Solid, m.p:145 °C ^1^H NMR (600 MHz, CDCl_3_): ∂ 3.90 (s, 6H, OCH_3_), 3.97 (s, 3H, OCH_3_), 7.18 (s, 2H, Ar–CH), 7.65 (s, 1H, CH=(CN)_2_).^13^C NMR (150 MHz, CDCl_3_): ∂ 56.37, 61.30, 80.60, 108.26, 113.23, 114.02, 125.96, 143.97, 153.37, 159.45; ATR-IR: 2942, 2839, 2221, 1568, 1499, 1455, 1247, 1126.92; MS (*m/z*) for C_13_H_12_N_2_O_3_ (M−H)^+^: Caled: 243.1, Found: 243.

### Computational methods

All calculations are performed using Gaussian 09 W [21] program package. In the present work, B3LYP/6–311++G** level of theory is employed to achieve our aim from this study. Becke’s three parameter hybrids function combined with the Lee–Yang–Parr correlation function (B3LYP) [31–34] predict the best results for molecular geometry and electronic transition for moderately larger molecules. B3LYP/6–311++G** frequency analysis calculations were performed to characterize the stationary points as the minima. HOMO–LUMO energies, absorption wavelengths and oscillator strengths are calculated using TD-B3LYP [35–37]. These optimized structures were calculated for the first excitation energy, maximal absorption wavelength (λ_max_) and oscillator strengths (f) for the three states by using TD-B3LYP/6–311++G** level of theory. Moreover, three density functional, namely, PBEPBE [38] with same above basis set have been evaluated in order to find out the suitable functional that estimates the absorption behavior of the studied dyes.

## Conclusions

In this paper, different donor-π-acceptor compounds having dicyanovinyl as the acceptor and aryl moieties as donors were synthesized. Compared with all molecules investigated, molecule 5 showed the highest Stokes shift as well as the highest fluorescent intensity indicating a typical molecular rotor. Also, the energy Eg values were nicely correlated with the donor ability of the substituent as presented by Hammett resonance effect. UV–visible absorption maxima of the compounds were examined experimentally as well as computationally and the results obtained have shown that TD-DFT calculations, with a hybrid exchange–correlation and the long-range corrected density functional PBEPBE with a 6–311++G** basis set, was reasonably capable of predicting the excitation energies, the absorption and the emission spectra of these molecules.
